# Association of Diabetic Retinopathy With Stroke: A Systematic Review and Meta-Analysis

**DOI:** 10.3389/fneur.2021.626996

**Published:** 2021-03-16

**Authors:** Kaiyan Hu, Mengyao Jiang, Qi Zhou, Weiting Zeng, Xuhong Lan, Qianqian Gao, Fan Mei, Li Zhao, Fei Chen, Anhu Wu, Gongcai Tao, Chenghua Mou, Bin Ma

**Affiliations:** ^1^Evidence-Based Medicine Centre, School of Basic Medical Sciences of Lanzhou University, Lanzhou, China; ^2^Evidence-Based Nursing Center, School of Nursing, Lanzhou University, Lanzhou, China; ^3^The First Clinical Medical College, Lanzhou University, Lanzhou, China; ^4^Zhongshan Ophthalmic Center, Sun Yat-Sen University, Guangzhou, China; ^5^Department of Critical Care Medicine, Gansu Provincial Hospital, Lanzhou, China; ^6^The Second Clinical Medical College, Lanzhou University, Lanzhou, China

**Keywords:** diabetic retinopathy, stroke, systematic review, meta-analysis, epidemiology

## Abstract

**Background:** The population-based studies conducted thus far do not provide conclusive evidence of the link between diabetic retinopathy (DR) and stroke. The aim of the present systematic review was to determine whether DR is specifically associated with stroke.

**Methods:** MEDLINE, Embase, and Web of Science were systematically searched from their inception to July 31, 2020. All cohort studies that reported associations between the presence of DR and incident stroke were included. The pooled hazard ratios (HRs), pooled risk ratios (RRs), and 95% confidence intervals (CIs) were calculated.

**Results:** The meta-analysis included 19 cohort studies involving 81,452 diabetic patients. The pooled effect size of any DR related to stroke was 1.25 for HR (95% CI: 1.12–1.39; *P* < 0.0001) and 1.96 for RR (95% CI: 1.60–2.39; *P* < 0.0001). Subgroup analysis for the type of diabetes yielded pooled HR of 1.29 (95% CI: 1.10–1.50; *P* = 0.001) in patients with type 2 diabetes mellitus (T2DM). The pooled RR was 2.29 (95% CI: 1.77–2.96; *P* < 0.0001) in patients with T2DM. Two studies addressed the DR-related stroke among type 1 diabetes mellitus (T1DM) patients. One study found a significant association between DR and stroke (OR: 1.6; 95% CI: 1.1–2.3; *P* < 0.01), while the other did not identify an association between these two conditions (RR: 1.40; 95% CI: 0.62–2.18; *P* = 0.178).

**Conclusions:** The presence of DR is associated with an increased risk of stroke in diabetic patients. This correlation is robust in T2DM patients but uncertain in T1DM patients. Our findings indicate that DR is an important biomarker for the prediction of stroke. To further validate the role of DR in stroke-risk stratification, additional research is required on the association between the stage of DR and stroke risk, and more studies including T1DM patients are necessary.

## Introduction

Diabetic retinopathy (DR) is a common and specific microvascular complication of diabetes. Although largely preventable, DR affects a third of diabetic patients (1) and is the leading cause of vision loss in working-age individuals (2). The evidence is growing that DR reflects systemic microcirculatory disease affecting not only the eye but also other vital organs (3). The presence of DR signifies an increased heightened risk of life-threatening systemic vascular complications (3). Retinopathy was proposed to represent a novel biomarker of the risk for vascular disease patients with diabetes due to its specificity and the possibility of a non-invasive assessment (4). Evidence from meta-analyses documented that both early and advanced stages of DR are linked to macrovascular complications and all-cause mortality of diabetic patients (5, 6). However, no meta-analysis has been performed to specifically determine the relationship between DR and the individual clinical manifestations of cardiovascular disease (CVD), and the existing population-based studies did not provide conclusive evidence of the link between DR and stroke (7).

Worldwide, stroke constitutes a major health and societal burden for patients and their families (8). Given limited therapeutic options, effective preventive strategies (9) and methods for early diagnosis (10) are needed. The clinical symptoms of stroke manifest late in the disease course, but the underlying subclinical pathological processes take place much earlier (10). The retina and the brain share similar embryological origin, anatomical features, and physiological properties. Therefore, the retina offers a unique and easily accessible “window” to study the correlates and consequences of subclinical pathological changes underlying stroke (11). Vascular lesions seen in the eyes of patients affected by DR, such as microaneurysms, exudates, hemorrhages, and cotton wool spots, may mirror similar pathological processes occurring in the cerebral microcirculation (3). Therefore, understanding the relationship between DR and stroke has important implications for DR screening, the management of risk factors for stroke in diabetic patients, and the prediction of stroke risk.

The aim of the present study was to determine whether DR is specifically associated with any stroke and with specific subtypes of stroke by conducting a comprehensive systematic review and meta-analysis. Additionally, the association between DR severity or DR lesions and stroke was also evaluated.

## Methods

The study design and reporting of data are compliant with the Meta-analysis of Observational Studies in Epidemiology (MOOSE) guidelines (12) (Supplementary Table 1). The protocol for this systematic review was registered at the International Prospective Register of Systematic Reviews (PROSPERO); the registration number is CRD42020202571.

### Eligibility Criteria

The eligibility criteria related to study characteristics were as follows: (1) study design: population-based cohort studies or randomized controlled trials (RCTs) reporting an association between the presence of DR and incident stroke event; (2) participants: patients with type 1 or type 2 diabetes, regardless of age, race, and region; (3) exposure: DR was diagnosed by a reliable technology (e.g., retinal photography, fluorescein angiography) and the degree and/or severity was defined according to well-validated scales [e.g., Early Treatment Diabetic Retinopathy Study (ETDRS) adaptation of the modified Airlie House Classification of DR (13), International Clinical Diabetic Retinopathy Severity Scale (14)]; and (4) outcome: fatal or non-fatal stroke event. The stroke was defined as either a clinically diagnosed stroke or transient ischemic attack (with or without cerebral imaging).

The eligibility criteria related to the report characteristics were as follows: (1) publication language: English and (2) publication status: abstracts of studies were excluded.

### Data Sources and Searches

Two reviewers (K-YH and M-YJ) independently searched the MEDLINE (*via* PubMed), Embase, and Web of Science databases from their inception to July 31, 2020. The following terms were used: “stroke,” “cerebrovascular accident,” “apoplexy,” “hemiplegia,” “paresis,” “transient ischemic attack,” “cerebral,” “cerebellar,” “brain,” “vertebrobasilar,” “subarachnoid,” “infarct,” “ischemia,” “thrombosis,” “emboli,” “hemorrhage,” “hematoma,” “bleed,” “diabetic retinopathy,” “NPDR,” and “PDR.” The search strategy is detailed in Supplementary Table 2. To identify potential additional studies, we also searched Google Scholar, relevant reviews, and the references cited in included studies.

### Study Selection

All search results were exported to the EndNote X8 software for the removal of duplicates. Two reviewers (K-YH and M-YJ) independently screened titles and abstracts based on the eligibility criteria. Prior to the formal selection of studies, a random sample of 10% of records was independently evaluated by the two reviewers, and the final selection process did not commence until a satisfactory agreement (>90%) was achieved between them. Studies were subcategorized into three groups (included, excluded, and unsure) in this step. Then, two reviewers independently examined the full text of potentially eligible and unclear studies to reach the final decision of inclusion or exclusion. Any disagreements between the two reviewers were resolved by discussion or through consultation with a third author (BM).

### Data Extraction

Two reviewers (K-YH and QZ) independently extracted relevant data using a standardized, predefined data collection form prepared using Microsoft Excel 2016. Extracted data were reviewed and cross-checked by the two reviewers prior to cleaning and analysis. Any disagreements were solved by discussion or through consultation with a third author (W-TZ). Before the final extraction, a pretest using a random sample of five included studies was carried out to revise the form, and its final version was consulted with clinicians.

The extracted information included the following: (1) general characteristics of the study: first author, country, year of publication, study design, number of study centers, the origin of the cohort, setting, sample size, and follow-up period; (2) general characteristics of the subjects: age, gender, diagnostic criteria of diabetes, diabetes type, duration of diabetes, diabetes treatment, hypertension, systolic blood pressure, diastolic blood pressure, total cholesterol level, triglycerides, high-density lipoprotein cholesterol (HDL-cholesterol), low-density lipoprotein cholesterol (LDL-cholesterol), previous stroke, atrial fibrillation, and current smoking; (3) details of the exposure: the prevalence of DR and the method of DR identification; (4) study outcomes: method of stroke event identification, stroke type, and stroke event incidence; (5) effect size: hazard ratio (HR), risk ratio (RR), odds ratio (OR), and 95% confidence intervals (CI), as well as their adjustment factors. When several adjusted values of the effect size were available in a study, the most adjusted estimate was extracted. For duplicated study populations, the one with the longest follow-up or with the largest sample size was selected. Corresponding authors were contacted when it was not possible to extract the necessary data from a published paper.

### Assessment of the Risk of Bias

Two reviewers (K-YH and QZ) independently evaluated the risk of bias in the study using the Newcastle Ottawa Scale (NOS) (15), which consists of three parameters of quality: selection, comparability, and exposure assessment. Studies scoring >7 were considered to have a low risk of bias, scores of 5–7 indicated a moderate risk of bias, and scores of <5 indicated a high risk of bias. Any disagreement between two reviewers in the quality assessment was resolved by discussion or consultation with a third author (BM).

### Statistical Analysis

The pooled risk estimate of HRs, RRs, and their 95% CIs extracted from the included studies or calculated from the extracted data were examined to summarize stroke event associated with DR. If stroke events were rare (incidence <5%), OR and RR were considered to be equivalent (16); otherwise, OR was converted to RR for data synthesis (16). Statistical heterogeneity was assessed by the forest plot and tested using the χ^2^ and *I*^2^ method. In the absence of statistical heterogeneity [*P* > 0.10 and *I*^2^ < 50% (17)] among the results, the meta-analysis was performed using the Mantel–Haenszel statistical method with the fixed-effect model. In the presence of statistical heterogeneity [*P* < 0.10 and *I*^2^ > 50% (17)] among the results, the Mantel–Haenszel statistical method with the random effect model was used for meta-analysis. We planned to conduct subgroup analyses using the following factors: type of diabetes [type 1 diabetes mellitus (T1DM) vs. type 2 diabetes mellitus (T2DM)], subtypes of stroke (ischemic vs. hemorrhagic), and subtypes of ischemic stroke (lacunar vs. cortical). When 10 or more studies were included (18) in each outcome, metaregression analyses were used to explore the source and size of heterogeneity in a study and to explain the impact of heterogeneity in the meta-analysis. If necessary, sensitivity analyses were performed by excluding studies with a high risk of bias and studies with a small sample size. Publication bias was assessed using funnel plots and Egger's regression tests when at least 10 studies were available (19). The level of statistical significance was set at *P* < 0.05 using two-sided tests. The Stata 12.0 software was used to perform data synthesis.

## Results

### Literature Search and Selection Results

**Figure 1:**
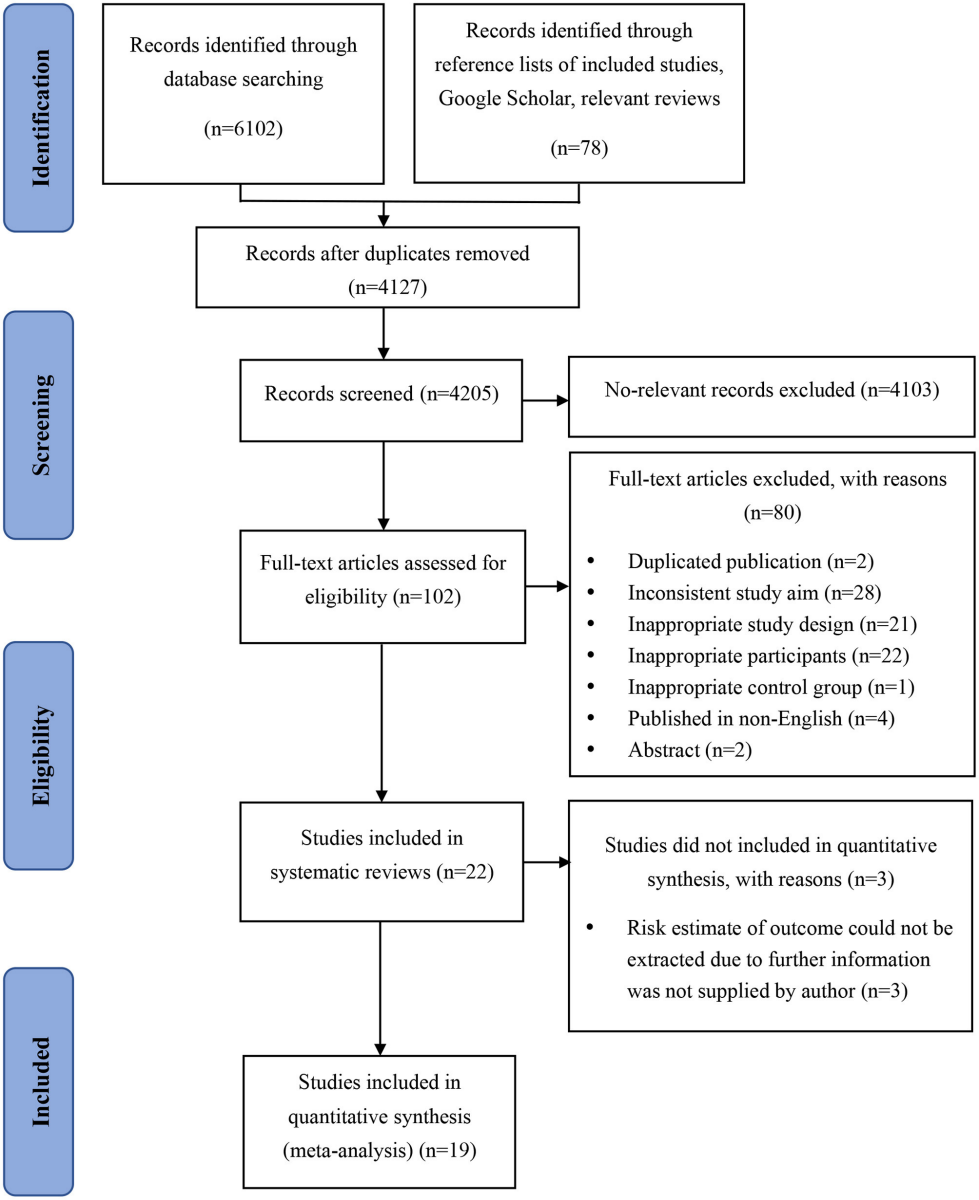
Flowchart of the selection of studies.

The search of electronic databases identified 4,127 studies after duplicates were removed. A supplementary search conducted on August 1, 2020, identified 78 additional potentially relevant studies. The study selection process is presented in Figure 1. After detailed assessment based on eligibility criteria, 22 studies were included in the review, of which 3 did not report risk estimate or stroke event incidence, necessitating to contact the authors to obtain these further data. Since no reply was received from the authors of two studies (20, 21) after three contact attempts, and the author of one study (22) replied that they did not have the resources at that time to rerun the models, these three publications have been excluded. Finally, 19 studies (23–41) were included in the present meta-analysis.

### Characteristics of Studies and Participants

The characteristics of the 19 studies are shown in Table 1, and additional information is provided in Supplementary Table 4. In 12 studies (23–27, 29–31, 33, 38, 40, 41), the risk of bias was rated as low, and the remaining seven studies (28, 32, 34–37, 39) were rated as having a moderate risk of bias (Supplementary Table 5). Eleven publications (23–31, 36, 37) reported large population-based studies, two (38, 41) were hospital-based studies, and six originated from within a clinical trial (32–35, 39, 40). These studies were performed in the USA, UK, Australia, Japan, China, Canada, Sweden, and Greece.

**Table 1:** Characteristics of the studies included in the meta-analysis.

**Study (year)**	**Country**	**Study design**	**Follow-up period, years**	**Sample size (% males)**	**Age, years**	**Diabetes type**	**DR identification**	**Stroke identification**	**Stroke type**	**Event (*n*)**	**Main findings reported in original studies: OR/RR/HR (95% CIs)**		**Adjustment factors**
Cheung (2006)	Australia	Prospective cohort	Mean ± SD: 7.8 ± 1.9	1,546 (47.0)	Range: 45–64	Both	Fundus photograph and ETDRS adaptation of the modified Airlie House Classification	Medical records	Ischemic stroke	Non-fatal stroke (75)	HR	Any DR: 2.34 (1.13–4.86); severe NPDR: 1.81 (0.57–6.4); retinal hemorrhages or microaneurysms: 1.33 (0.45–2.21); hard exudates: 1.45 (0.61–3.43); cotton wool spots: 2.10 (0.90–4.91)	Age, sex, race, and examination center, 6-year mean arterial blood pressure, use of antihypertensive treatment, fasting glucose, use of insulin, duration of diabetes, high-density lipoprotein and low-density lipoprotein cholesterol, and cigarette smoking status
Fuller (2001)	UK	Prospective cohort	12	4,743 (48.9)	Range: 35–53	Both	Fundus photograph and self-defined classification^$^	Self-reported diagnosis history/medical records /death certificate	NR	Fatal or non-fatal stroke (293)	RR	T1DM: 1.4 (0.62–2.18); T2DM: 2.25 (1.61–2.89)	Age
Landers (2018)	Australia	Prospective cohort	Median: 8.7	1,257 (37.9)	Range: ≥40	Both	Slit-lamp fundoscopy and ETDRS adaptation of the modified Airlie House Classification	Medical records	NR	Fatal stroke (NR)	HR	5.81 (1.21–27.88)	Age, sex, and presence of systemic hypertension
Drinkwater (2020)	Australia	Prospective cohort	Mean ± SD: 6.6 ± 1.8	1,473 (51.9)	NR	T2DM	Fundus photograph and ETDRS adaptation of the modified Airlie House Classification	Medical records/death certificate/autopsy findings	Ischemic/ hemorrhagic/ unspecified stroke	Fatal or non-fatal stroke (53)	HR	Any stroke: 2.19 (0.79–6.07); ischemic stroke: 1.13 (0.49–2.63); hemorrhagic stroke: 0.36 (0.08–1.68); moderate NPDR or worse vs. mild or no NPDR: 2.55 (1.19–5.47)	Age, sex, duration of diabetes, diabetes treatment, blood pressure, HbA1c, BMI, urinary albumin:creatinine ratio, lipids, smoking status, atrial fibrillation, macroalbuminuria
Kawasaki (2013)	Japan	Prospective cohort	8	1,620 (53.7)	Mean ± SD: 58.3 ± 7.0	T2DM	Ophthalmoscopic examination and slit-lamp biomicroscopic fundus examination/ fundus photography/ fluorescein angiography and international clinical diabetic retinopathy severity scales	Death certificates/ medical records/ self-reported diagnosis history	Ischemic/ hemorrhagic/ unspecified stroke/ TIA	Fatal or non-fatal stroke (76)	HR	Any DR: 1.69 (1.03–2.8); moderate NPDR: 2.15 (0.75–6.21); retinal hemorrhages or microaneurysms: 1.63 (0.97–2.73); hard exudates: 1.76 (0.62–4.97); cotton wool spots: 2.39 (1.35–4.24)	Age, sex, hemoglobin A1c, duration of diabetes, body mass index, systolic blood pressure, low-density lipoprotein cholesterol, log triglycerides, log albumin-to-creatinine ratio, and smoking
Klein (2004)	USA	Prospective cohort	20	996 (46.8)	NR	T1DM	Fundus photograph and ETDRS adaptation of the modified Airlie House Classification	Self-reported diagnosis history/medical records	NR	Non-fatal stroke (55)	OR	1.60 (1.10–2.30)	Age, sex, glycosylated hemoglobin, hypertension, neuropathy, daily aspirin, ocular factors
Klein (1999)	USA	Prospective cohort	Median: 16	1,370 (46.4)	NR	Both	Fundus photograph and ETDRS adaptation of the modified Airlie House Classification	Death certificate	NR	Fatal stroke (175)	HR	PDR: 1.88 (1.03–3.43)	Age, sex, glycosylated hemoglobin, hypertension, urine protein, history of cardiovascular disease
Chou (2016)	China	Retrospective cohort^*^	12	37,816 (49.0)	NR	Both^a^	Medical records	Medical records	Ischemic stroke	Non-fatal stroke (4,698)	HR	1.114 (0.888–1.23)	Age, sex, hypertension, heart failure, previous stroke/TIA, previous vascular diseases, end-stage renal disease, COPD, malignancy, autoimmune disorders, liver cirrhosis, sleep apnea, sulfonylureas, meglitinide, metformin, AGI, insulin, ARB, ACEI, thiazides, calcium channel blockers, statins, beta blocker
Petitti (1995)	USA	Retrospective nested case–control	12	2,124 (52.0)	Mean: 67	Both	Medical records	Medical records	Ischemic stroke (non-embolic)	Non-fatal stroke (52)	RR	4 (1–14.5)	Age, sex, smoking, insulin, average systolic blood pressure, average random glucose, any other complication
Seferovic (2018)	USA	RCT	Range: 0.83–3.91	6,068 (69.3)	Mean ± SD: 60.3 ± 9.7	T2DM^b^	Self-reported diagnosis history	Clinical diagnosis	Ischemic/ hemorrhagic/ unspecified stroke	Non-fatal stroke (127)	HR	1.28 (0.075–2.19)	Age, sex, race, BMI, baseline HbA1c, smoking, history of hypertension, heart rate, total cholesterol, low-density lipoprotein cholesterol, and triglycerides, neuropathy, T2DM duration, and randomized study treatment
Gerstein (2013)	Canada	RCT	Mean: 4	2,856 (61.8)	NR	T2DM	Fundus photographs and modified version of the ETDRS Final Diabetic Retinopathy Severity Scale	Clinical diagnosis/autopsy	Ischemic/ hemorrhagic stroke	Fatal or non-fatal stroke (32)	HR	Any DR: 1.34 (0.78–2.3); severe NPDR: 2.05 (0.88–4.8)	The clinical center network, cardiovascular event prior to randomization (i.e., secondary prevention), blood pressure trial, intensive glycemia group, intensive blood pressure group, and fibrate group
Hitman (2007)	UK	RCT	Median: 3.9	2,778 (69.3)	Range: 40–75	T2DM	Medical records/ self-reported any retinopathy, maculopathy or previous photocoagulation	Clinical diagnosis/autopsy	Ischemic/ hemorrhagic/ unspecified stroke	Fatal or non-fatal stroke (NR)	HR	1.72 (1.03–2.87)	Unadjusted
Hankey (2013)	Australia	RCT	Median: 5	9,795 (62.6)	Range: 50–75	T2DM	Self-reported diagnosis history	clinical diagnosis	Ischemic/ hemorrhagic/ unspecified stroke	Non-fatal stroke (333)	HR	Small artery ischemic stroke: 1.82 (1.08–3.07); large artery ischemic stroke (negative results not reported)	Randomized study treatment
Hjelmgren (2019)	Sweden	Retros -pective cohort	Median: 3, IQR: 1–5	445 (64.7)	Range: ≥40	T2DM^c^	Fundus photograph and medical records (fundus photograph show signs of DR)	Medical records/ death certificate	Ischemic stroke	Fatal or non-fatal stroke (62)	HR	0.89 (0.51–1.53)	Age, coronary heart disease, heart failure, periphery artery disease and creatinine
Su (2017)	China	Retros -pective cohort^*^	Median: 5.21	755 (NR)	Range: ≥20	Both	Medical records	Medical records	Ischemic/ hemorrhagic stroke	Non-fatal stroke (NR)	HR	1.69 (1.05–2.72)	Neovascular glaucoma, age, sex, medical comorbidity, ocular comorbidity
Protopsaltis (2007)	Greece	Prospective cohort	Median: 10.1, IQR: 8.2–13.4	599 (45.7)	Mean ± SD: 60.4 ± 9.6	T2DM	NR	Medical records/ self-reported disease history	Ischemic stroke (non-embolic)	Non-fatal stroke (78)	HR	1.297 (0.816–1.61)	Age, gender, smoking, BMI, HbA1c, lipids, and diabetes duration
Bello (2014)	USA	RCT	Mean: 2.4	4,038 (42.7)	NR	T2DM^d^	Self-reported diagnosis history	Clinical diagnosis	Ischemic/ hemorrhagic/ unspecified stroke	Fatal stroke (NR)	HR	0.96 (0.7–1.32)	Unadjusted
Cohen (2003)	USA	RCT	Mean: 5.3	950 (60.9)	Range: 40–74	T2DM	Fundus photograph and ETDRS adaptation of the modified Airlie House Classification	Clinical diagnosis	NR	Fatal or non-fatal stroke (41)	RR^f^	2.16 (1.1–4.28)	Unadjusted
Ono (2002)	Japan	Prospective cohort	Mean ± SD: 11.6 ± 4.9	223 (77.1)	NR	T2DM^e^	Ophthalmologic records including ophthalmologic charts, fundus photography, and fluorescein retinal angiography and modification of the Diabetic Retinopathy Study and the ETDRS	Medical records/self-reported diagnosis history	NR	Fatal stroke (9)	RR^f^	2.28 (0.63–8.24)	Unadjusted

*USA, the United States of America; UK, the United Kingdom; RCT, randomized controlled trial; NR, no report; SD, standard deviation; T2DM, type 2 diabetes mellitus; T1DM, type 1 diabetes mellitus; TIA, transient ischemic attack; ETDRS, Early Treatment Diabetic Retinopathy Study; DR, diabetic retinopathy; PDR, proliferative diabetic retinopathy; NPDR, non-proliferative diabetic retinopathy; HR, hazard ratio; RR, risk ratio; OR, odds ratio; HbA1c, hemoglobin A1c; BMI, body mass index; COPD, chronic obstructive pulmonary disease; AGI, alpha-glucosidase inhibitors; ARB, angiotensin II receptor blockers; ACEI, angiotensin-converting enzyme inhibitors*.

$ *NPDR: one or more microaneurysms or hemorrhages with and without soft or hard exudates; PDR: definite retinal neovascularization, vitreous hemorrhages, or history of photocoagulation treatment; any retinopathy: either NPDR or PDR*.

* *Data originated from a national database*.

a *Diabetes patients with atrial fibrillation*.

b *Diabetes patients with acute coronary syndrome*.

c *Diabetes patients all suffered their first stroke or TIA*.

d *Diabetes patients with chronic kidney disease and moderate anemia*.

e *Diabetes patients with multivessel coronary artery disease following coronary artery bypass graft surgery*.

f *Calculated from raw data*.

The 19 studies included 81,452 diabetic patients. In the 13 studies that provided this information, the age of patients varied from 20 to 75 (34, 37). The gender of patients was extracted from 18 publications that provided this information. Among these studies, the fraction of males per studies varied from 37.9 to 77.1%. Ten studies (26, 27, 32–36, 38–41) included only T2DM patients, one study (28) included only T1DM patients, and the remaining eight studies (23–25, 29–31, 37) included patients with both types of diabetes.

The retinal assessment techniques were well-described and performed in most studies. Eight of them performed retinal photography (23, 24, 26, 28, 29, 33, 36, 40); one performed direct ophthalmoscopy (25); two performed retinal photography, direct ophthalmoscopy, or fluorescein angiography (27, 41); three relied on medical records (30, 31, 37); three utilized self-reported diagnostic history (32, 35, 39); and one used medical records, self-reported diagnosis, or treatment history (34). One study did not record the method of retinal assessment (38). Nine of the 19 studies used DR classification scales that had been well-validated either internally or externally (23, 25–29, 33, 40, 41).

The definition of stroke was similar in all studies. Thirteen of them defined “stroke” in the index paper or related publications. All these 13 studies (23, 26, 27, 30–39) assessed some measures of clinical stroke, and one also included transient ischemic attack (TIA) (27). Eight studies (26, 27, 32–35, 37, 39) included patients with any type of stroke, and two of these studies (26, 35) subtyped stroke into hemorrhagic or ischemic, with one further subtyping the ischemic stroke into large artery ischemic stroke and small artery ischemic stroke (35). The remaining five studies (23, 30, 31, 36, 38) only included ischemic stroke, and two of them (31, 38) only included non-embolic stroke.

The methods used for stroke identification varied widely. Four studies (32, 35, 39, 40) used examination by a specialist at the time of stroke; two studies (33, 34) relied on clinical assessment by study investigators, but not necessarily at the time of the stroke; and 13 studies (23–31, 36–38, 41) used the self-reported history of diagnosis, review of medical records, or death certificates. The incidence of stroke varied from 1.1 to 13.9% in 16 studies (23, 24, 26–36, 38, 40, 41) that provided this information. Among the 19 included studies, eight reported non-fatal stroke only (23, 28, 30–32, 35, 37, 38), four reported fatal stroke only (25, 29, 39, 41), and the remaining seven reported both fatal and non-fatal stroke (24, 26, 27, 33, 34, 36, 40).

Fourteen studies (23, 25–27, 29, 30, 32–39) reported HR as effect size. One study (28) reported OR as effect size. Two studies (24, 31) reported RR as effect size, and two (40, 41) reported only the incidence of stroke in the exposure and control groups, from which we calculated the crude RR for data synthesis. In 15 of the 19 studies (23–33, 35–38), the data were adjusted appropriately.

### Association Between DR and Stroke Event

#### Any DR and Stroke Event

**Figure 2:**
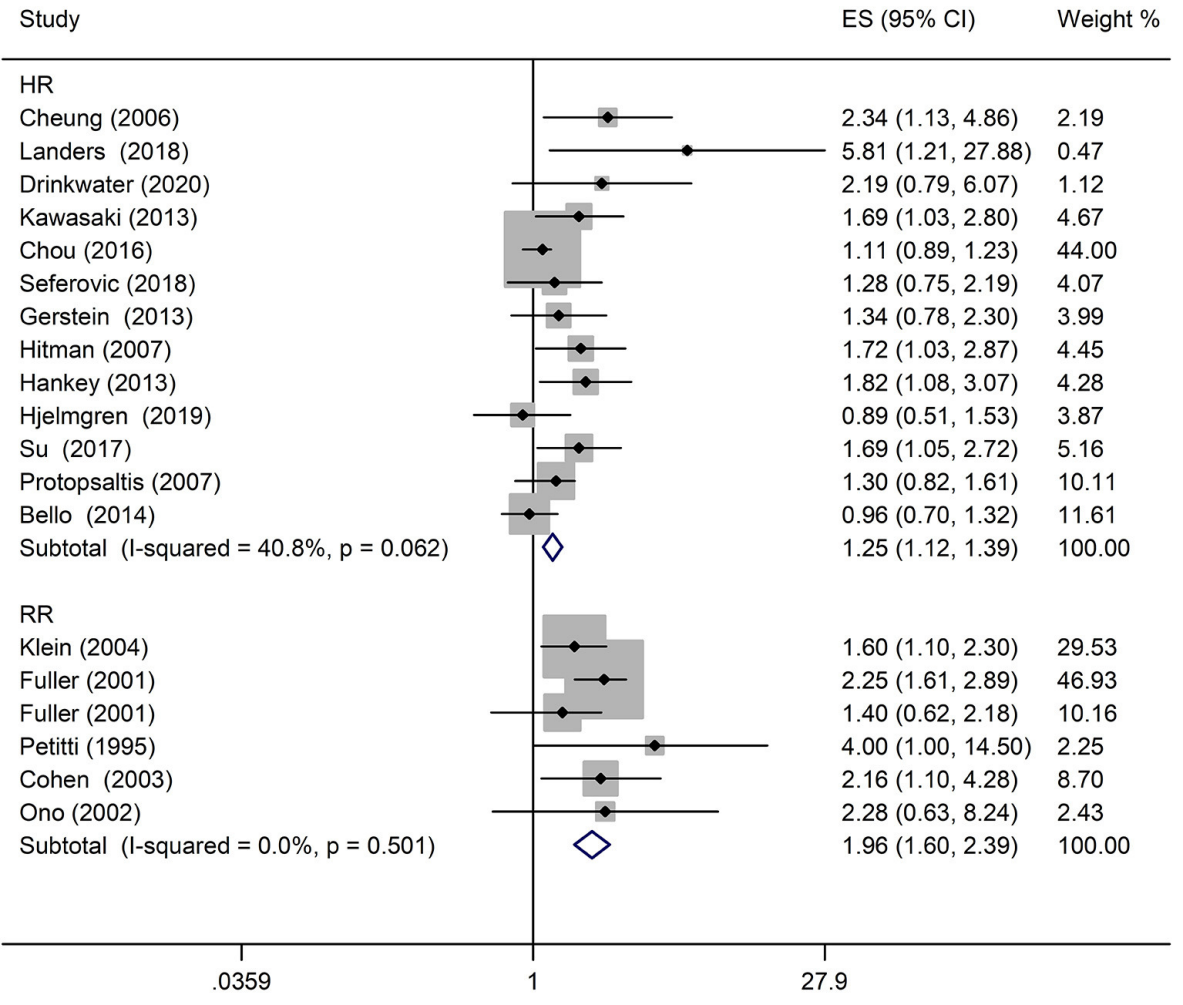
Pooled hazard ratio/risk ratio (HR/RR) for the association of any diabetic retinopathy (DR) with stroke event in patients with any type of diabetes.

**Figure 3:**
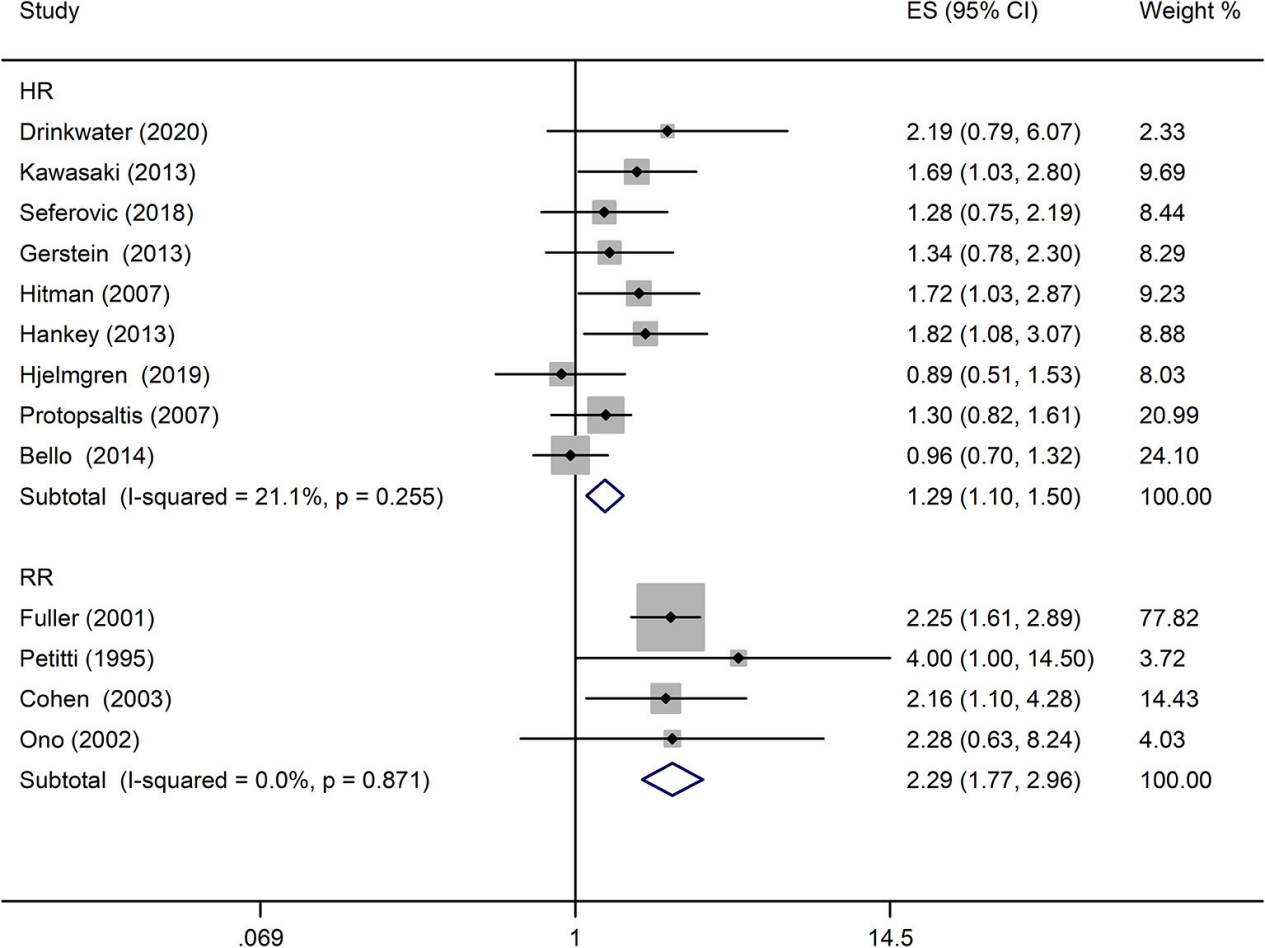
Pooled HR/RR for the association of any DR with stroke event in patients with T2DM.

**Figure 4:**
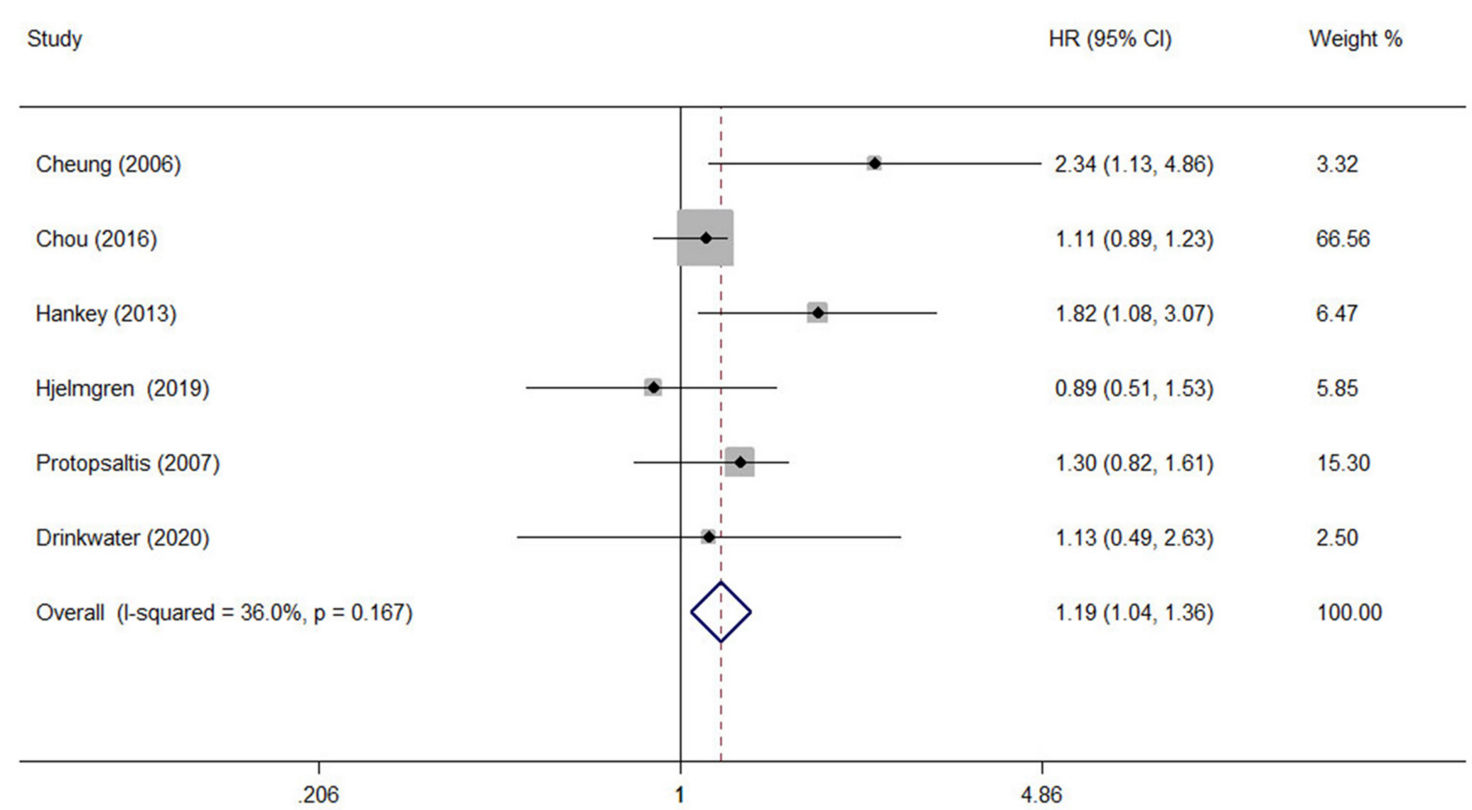
The pooled HR for the association of any DR with ischemic stroke type in diabetic patients.

A comprehensive analysis involving HR as an effect measure included 13 studies (23, 25–27, 30, 32–39). The pooled HR of any DR was found to be significantly associated with that of stroke event (HR: 1.25; 95% CI: 1.12–1.39; *P* < 0.0001; Figure 2). Eleven of these studies (23, 25–27, 30, 32, 33, 35–38) reported appropriately adjusted data. The median duration of the follow-up for 71,046 patients in the 13 studies was 5.21 years (interquartile range: 3.9–8 years). Since heterogeneity was low (*I*^2^ = 40.8%, *Q* = 20.27), the fixed-effects model was employed. Comprehensive analysis with RR as an effect measure included five studies (24, 28, 31, 40, 41). The pooled RR of any DR was found to be significantly associated with that of stroke event (RR: 1.96; 95% CI: 1.60–2.39; *P* < 0.0001; Figure 2). Since heterogeneity was low (*I*^2^ = 0.0%, *Q* = 4.34), the fixed-effects model was employed. Three of these five studies (24, 28, 31) reported appropriately adjusted data. The median duration of follow-up for 9,036 patients in the five studies was 12.00 years (interquartile range: 11.6–12.0 years). Metaregression analysis was performed to explore the source and size of heterogeneity. Our regression analysis showed that study design, diabetes type, and stroke identification method were not the sources of heterogeneity (Supplementary Table 3). However, due to missing information, other factors that might cause heterogeneity, such as follow-up period, mean age, the fraction of males, and geographic regions, have not been evaluated. The funnel plots and the Egger's test (Supplementary Figures 1, 2) showed that a significant publication bias (*P* = 0.01) was present in the association of any DR with stroke event in diabetes patients. Subgroup analysis for the type of diabetes yielded pooled HR of 1.29 (95% CI: 1.10–1.50; *P* = 0.001; Figure 3) and pooled RR of 2.29 (95% CI: 1.77–2.96; *P* < 0.0001; Figure 3) among patients with T2DM. Since heterogeneity was low (HR: *I*^2^ = 21.1%, *Q* = 10.14; RR: *I*^2^ = 0.0%, *Q* = 4.34), the fixed-effects model was employed. Two studies (24, 28) addressed the DR-related stroke among T1DM patients. One study (28) found a significant association between DR and stroke (OR: 1.6; 95% CI: 1.1–2.3; *P* < 0.01; Table 1), while the other (24) did not find such an association (RR: 1.40; 95% CI: 0.62–2.18; *P* = 0.178; Table 1). Subgroup analysis for stroke type yielded pooled HR of 1.19 (95% CI: 1.04–1.36; *P* = 0.01; Figure 4) for ischemic stroke. Since heterogeneity was low (*I*^2^ = 36.0%, *Q* = 7.81), the fixed-effects model was employed. Two studies (26, 35) analyzed the hemorrhagic subtypes of stroke and found that DR was not significantly associated with hemorrhagic stroke [HR: 0.36; 95% CI: 0.08–1.65; *P* = 0.188; one study (35) did not provide negative results; Table 1]. Only one study (35) subtyped ischemic stroke into large artery ischemic stroke and small artery ischemic stroke and determined that DR was significantly associated with small artery ischemic stroke (HR: 1.82; 95% CI: 1.08–3.07; *P* = 0.03, Table 1) but not associated with large artery ischemic stroke (no negative results reported, Table 1).

#### DR Severity and Stroke Event

**Figure 5:**
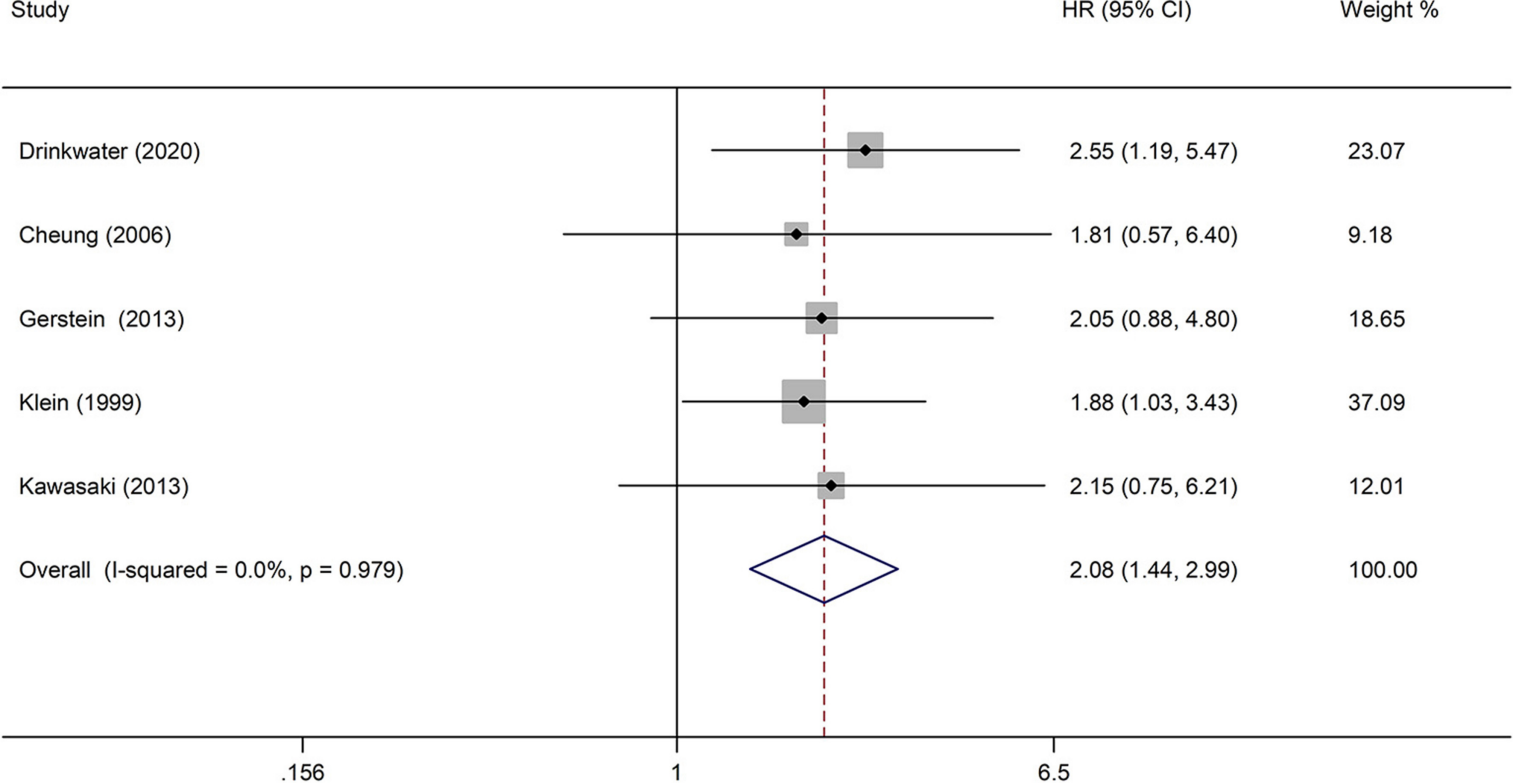
Pooled HR for the association of moderate NPDR or more severe DR with stroke event in diabetic patients.

The analysis of the effect of DR severity on stroke event included five studies (23, 26, 27, 29, 33). The pooled analysis revealed that the HR for stroke events in moderate non-proliferative diabetic retinopathy (NPDR) or more severe DR was 2.08 (95% CI: 1.44–2.99, *P* < 0.0001; Figure 5) when compared with individuals with mild NPDR or no DR. All studies reported appropriately adjusted data. The median duration of follow-up for 8,865 patients in the five studies was 7.80 years (interquartile range: 6.6–8.0 years). Since heterogeneity was low (*I*^2^ = 0%, *Q* = 0.44), the fixed-effects model was employed.

#### DR Lesions and Stroke Event

**Figure 6:**
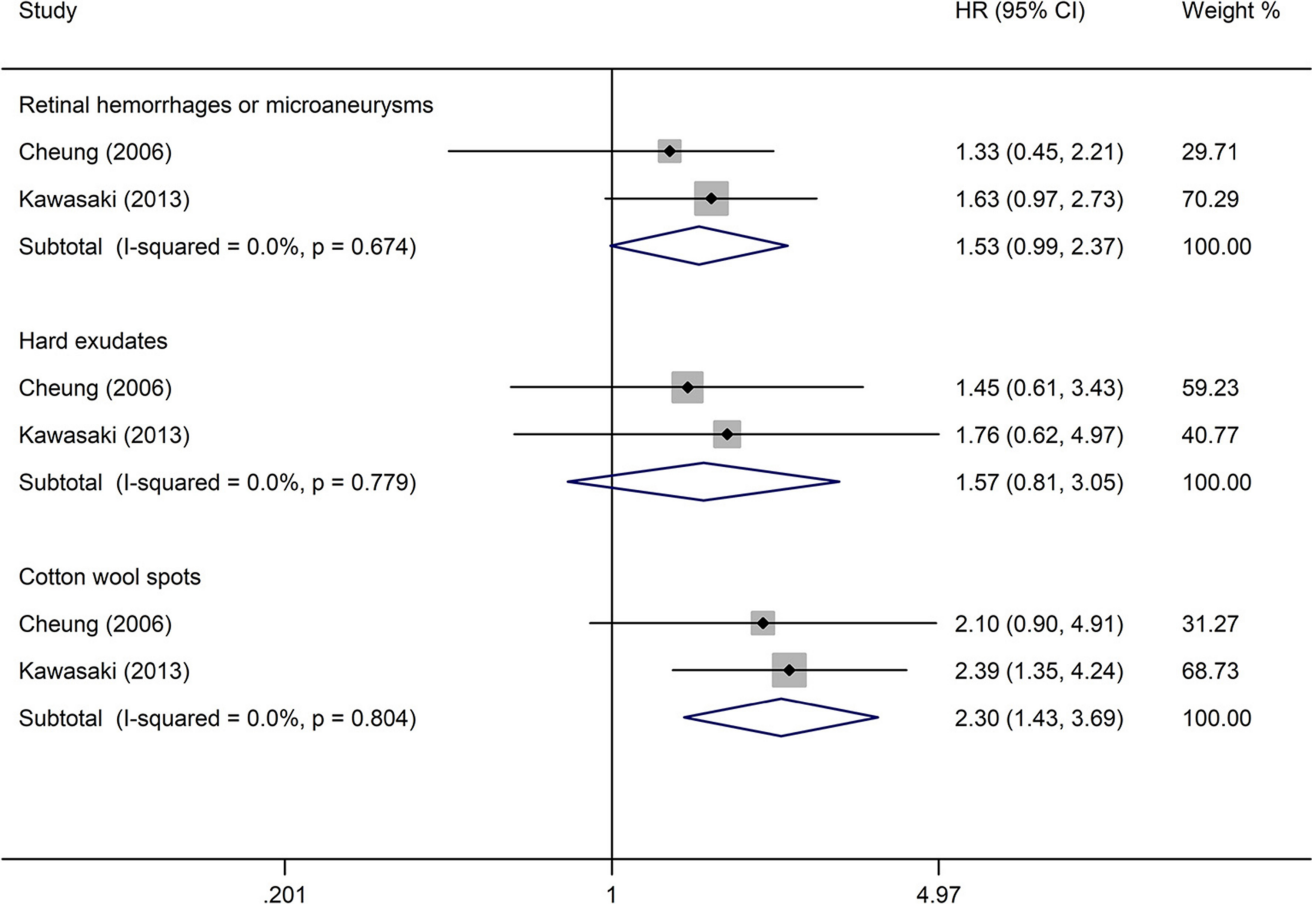
Pooled HR for the association of DR lesions with stroke event in diabetic patients.

The analysis of the effect of DR lesions on stroke event included only two studies (23, 27). The pooled HR of DR lesions related to stroke event in retinal hemorrhages or microaneurysms, hard exudates, and cotton wool spots was, respectively, 1.53 (95% CI: 0.99–2.37; *P* = 0.053; Figure 6), 1.57 (95% CI: 0.81–3.05; *P* = 0.184; Figure 6), and 2.30 (95% CI: 1.43–3.69; *P* = 0.001; Figure 6). All studies reported appropriately adjusted data. The median duration of follow-up for 3,166 patients in the two studies was 7.9 years (interquartile range: 7.8–8.0 years). Since heterogeneity was low (retinal hemorrhages or microaneurysms: *I*^2^ = 0%, *Q* = 0.18; hard exudates: *I*^2^ = 0%, *Q* = 0.08; cotton wool spots: *I*^2^ = 0%, *Q* = 0.06), the fixed-effects model was employed.

## Discussion

The performed meta-analysis, which included 81,452 participants across 19 cohort studies, demonstrated that the presence of any DR was associated with an increased risk for stroke event (fatal and non-fatal) in diabetic patients. This evidence was robust in T2DM patients but inconclusive in T1DM patients. When stroke subtypes were considered, robust evidence was found that any DR was significantly associated with ischemic stroke. Only one study considered the subtypes of ischemic stroke and found that any DR was associated with small artery ischemic stroke but not with large artery ischemic stroke. The association between DR and hemorrhagic stroke was not observed, but this finding was based on two studies only. Furthermore, we found a significant trend for the increased risk of stroke with increasing the stage of DR and severity of DR lesions.

The link between DR and stroke in diabetic patients can be explained by the fact that diabetes-related changes in the retinal microvasculature mirror those in the cerebral microvasculature (3, 7). Microvascular dysfunction is a widespread phenomenon in individuals with diabetes, including the effects on the brain (3, 7). The microvasculature of the retina and brain is closely linked, offering a unique opportunity to study microvascular changes in the brain because the retinal structures can be directly visualized (11), while direct evaluation of the brain microvasculature is impossible with current neuroimaging techniques. Another potential mechanism linking DR with stroke might be the common risk factors for DR and stroke, such as elevated hemoglobin A1c (HbA1c), hyperglycemia, hypertension, and dyslipidemia (42, 43). In the subgroup analysis, we observed a strong association between any DR and stroke in T2DM, but this association was uncertain in T1DM. This could be explained by shared risk factors for DR and stroke, such as poor glycemic control, high blood pressure, and dyslipidemia, which are more often present in T2DM than T1DM due to the relatively older age (44). However, the underlying mechanism of the difference between the effects of T2DM and T1DM needs to be further clarified. To better understand the pathophysiology of DR-related stroke, we conducted two subgroup analyses for subtypes of stroke and subtypes of ischemic stroke. The result suggested that DR was significantly associated with ischemic stroke. One study showed that DR was a predictor of small artery ischemic stroke but not large artery ischemic stroke. Moreover, two studies found DR was not associated with hemorrhagic stroke. These findings are consistent with the role of cerebral microvascular dysfunction in diabetes (7).

The detailed analysis of the association between DR and stroke documented that the risk of stroke was 2.08 times higher in patients with moderate NPDR or more severe DR than in subjects without DR, which was significantly higher than the risk of stroke in patients with any DR (HR 1.25, 95% CI: 1.12–1.39). This finding supports indirectly the possibility of an association between the severity of DR and higher risk of stroke. Another identified dose–response effect was represented by the association of a higher risk of stroke with increased severity of DR lesions. The meta-analysis based on two studies suggested that the presence of cotton wool spots was significantly associated with stroke, but the presence of retinal hemorrhages or microaneurysms, as well as the presence of hard exudates, was not significantly associated with stroke. This finding further confirmed that the increased level of DR is related to a higher risk of stroke to a certain degree because the classification of DR was based on the severity of DR lesions. Furthermore, the strong association between the presence of cotton wool spots and stroke further confirms the possibility that similar pathologic changes are present in the cerebral and retinal microcirculation in DR patients. Pathologically, cotton wool spots in the retina constitute a focal retinal capillary obstruction (45), and the ischemic change in the retina observed as cotton wool spots may reflect similar pathologic changes in the cerebral microcirculation, which can trigger an ischemic stroke.

The performed meta-analysis has shown that DR was significantly associated with stroke in diabetic patients. DR is an important biomarker predicting stroke, and retinal imaging techniques offer an excellent way to study the effects of microvascular dysfunction in diabetes on small cerebral vessels. The obtained data point to the need for a better stroke monitoring and follow-up in patients with DR. Additionally, they highlight the strength of using DR as a biomarker to predict stroke in a clinical setting. First, DR is characterized by long-term stability (27), and complete natural resolution after the initial diagnosis is unlikely. Second, as the marker of concomitant cerebral microangiopathy (3, 7), DR can be directly assessed by non-invasive visualization using retinal imaging techniques. The evaluation of DR can identify a specific subgroup of patients who could benefit from a more intensive and expensive brain imaging (11). Finally, given that assessment of DR is performed by ophthalmologists, sharing this information and using it proactively in stroke risk management will benefit both clinicians and patients by allowing better prediction of stroke with minimum additional effort and cost.

This is the first systematic review assessing the relationship between DR and stroke in a large number of diabetic patients. Our results provide the best current evidence for the association between DR and stroke and contribute to the debate on whether or not DR predicts the incidence of stroke. We found that the studies with contradicting results have the following common characteristics: the use of retrospective study design (30, 36) or the RCT post-analysis data without appropriate adjustments (33, 39), a small sample size (38, 41), an inaccurate method for DR identification (32), and heterogeneity of the included population (e.g., duration or type of diabetes, comorbidity), which may introduce bias to the studies' results and affect the internal validity of researches. This systematic review has certain limitations. First, most of the included studies identified stroke based on self-reported diagnosis history, review of medical records, or death certificates, rather than relying on an assessment by an expert done at the time of the stroke and supported by the presence of clinical features and appropriately timed brain imaging. Although being deemed acceptable for assessing the prevalence of stroke in epidemiological studies, this simplified approach reduces the accuracy of diagnosis (46, 47) and may be inadequate for detailed studies of pathophysiology. Second, studies differed in their inclusion/exclusion criteria, follow-up period, DR identification method, geographic regions, and adjusted factors. All these differences may account for the observed heterogeneity, which may reduce the reliability of our analysis. Third, we found publication bias based on funnel plot inspection and Egger's regression test results. Although we performed a comprehensive systematic search, studies published in non-English languages were excluded due to restricted resources. These limitations should be considered when interpreting the results.

Additionally, this systematic review highlighted several gaps in the knowledge to be filled by future research. First, our study did not provide robust evidence of the association between DR severity and stroke since only a small number of studies carefully characterized DR level and lesions. Therefore, further studies are required to analyze in more detail the association between these two complications and stroke by the use of reliable methods for retinal assessment and the determination of DR classification. These developments would contribute significantly to the validation of the effect of DR on stroke-risk stratification. Second, only two studies addressed the DR-related stroke among T1DM patients, and their conclusions were inconsistent. Typically, T1DM patients have a higher level of HbA1c because of the lack of insulin. A previous study showed that high HbA1c values accounted for up to 11% of the risk of developing DR (48), and a higher prevalence of DR has been estimated in T1DM patients than in T2DM patients (49). We believe that future investigations should pay more attention to the DR-related stroke risk in T1DM patients. Third, although we have addressed the pathophysiology of DR-related stroke, it should be noted that the analysis conducted here was based on only one study, and this is because most stroke remains a clinical diagnosis. Therefore, data obtained in longitudinal studies that subdivide ischemic stroke into lacunar and cortical will be important to clarify this issue.

## Conclusion

The presence of DR is associated with an increased risk of stroke in diabetic patients. The evidence of this association was robust in T2DM patients but uncertain in T1DM patients. Furthermore, with the increasing severity of DR lesions, a significant increasing trend in the risk of stroke is present. Our findings indicate that DR is an important biomarker for the prediction of stroke in clinical practice. To further validate the role of DR in stroke-risk stratification, additional research is required to explore the detailed association between DR stage and stroke risk, and more studies including T1DM patients are necessary.

## Data Availability Statement

The original contributions presented in the study are included in the article/Supplementary Material, further inquiries can be directed to the corresponding author/s.

## Author Contributions

BM, KH, and CM conceived and designed the research. WZ, QZ, and KH designed the data collection. MJ, QZ, KH, AW, and GT performed the data collection. QZ, KH, and QG performed the statistical analysis. KH, FM, LZ, FC, WZ, and BM interpreted the data. KH wrote the first draft of the manuscript. All authors revised the manuscript for important intellectual content and read and approved the final manuscript.

## Conflict of Interest

The authors declare that the research was conducted in the absence of any commercial or financial relationships that could be construed as a potential conflict of interest.
