# Supplementary material for: Association of Diabetic Retinopathy With Stroke: A Systematic Review and Meta-Analysis
Source: Front Neurol. 2021 Mar 16;12:626996. doi: 10.3389/fneur.2021.626996 (PMC8007918; doi:10.3389/fneur.2021.626996)
Supplement: Supplementary file 1 [file Data_Sheet_1.docx]

**Supplementary Figure 1 Evaluation of publication bias by f Egger’s test.**


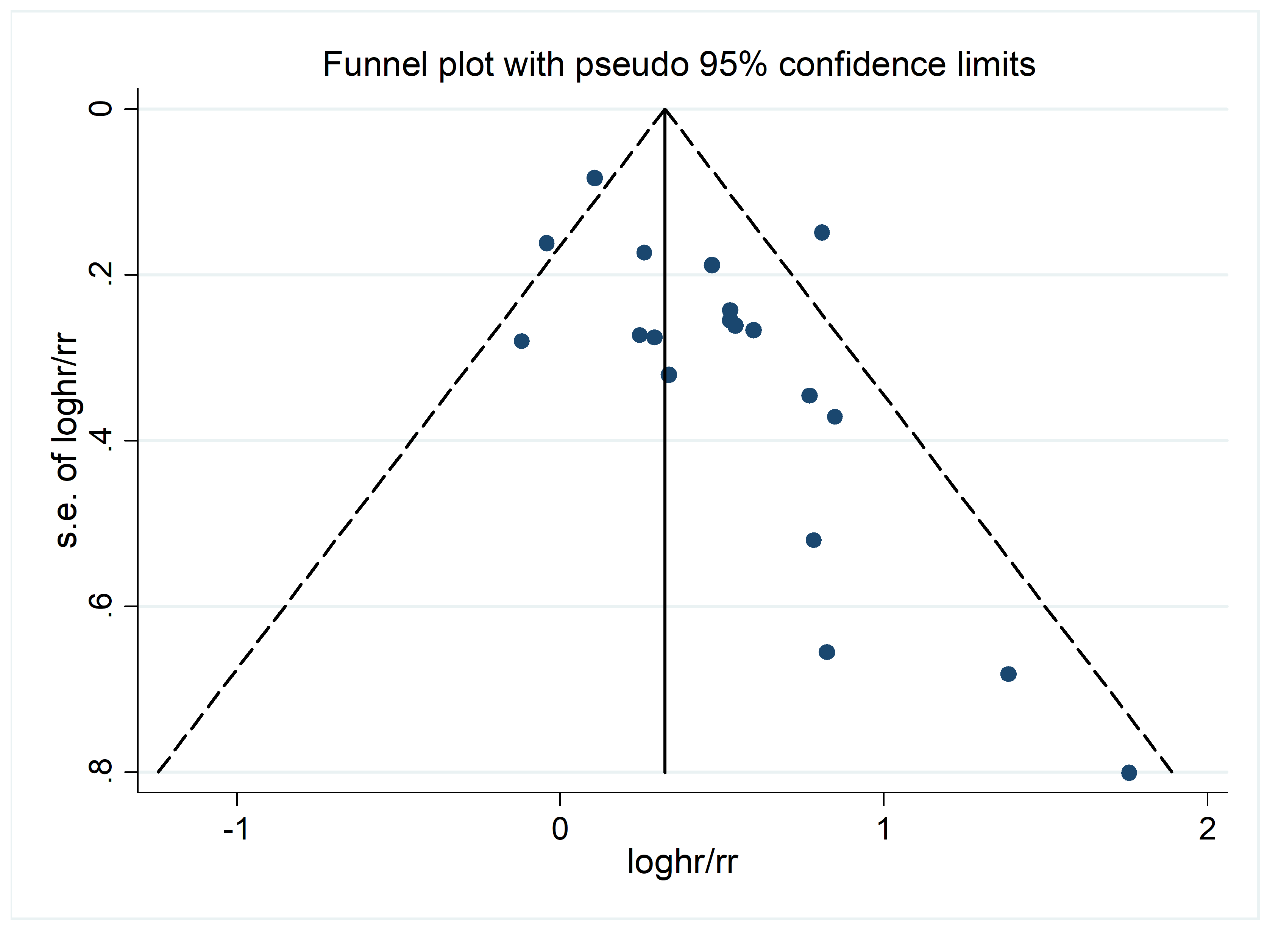


**Supplementary Figure 2 Funnel plot results of potential publication bias for the association of any DR with stroke event in diabetic patients.**

**Supplementary Table 1** **MOOSE checklist for meta-analyses of observational studies.**

| Item no. | Recommendation | Reported on page no. |
| --- | --- | --- |
| REPORTING OF BACKGROUND SHOULD INCLUDE | | |
| 1 | Problem definition | 2 |
| 2 | Hypothesis statement | 2 |
| 3 | Description of study outcome(s) | 2 |
| 4 | Type of exposure or intervention used | 2 |
| 5 | Type of study designs used | 2 |
| 6 | Study population | 2 |
| REPORTING OF SEARCH STRATEGY SHOULD INCLUDE | | |
| 7 | Qualifications of searchers (e.g., librarians and investigators) | 2 |
| 8 | Search strategy, including time period included in the synthesis and keywords | 2, Supplementary Table 2 |
| 9 | The effort to include all available studies, including contact with authors | 2 |
| 10 | Databases and registries searched | 2 |
| 11 | The search software used, name and version, including special features used (e.g., explosion) | 2 |
| 12 | Use of hand searching (e.g., reference lists of obtained articles) | 2 |
| 13 | List of citations located and those excluded, including justification | 2, Figure 1 |
| 14 | Method of addressing articles published in languages other than English | 2 |
| 15 | Method of handling abstracts and unpublished studies | 2 |
| 16 | Description of any contact with authors | 3 |
| REPORTING OF METHODS SHOULD INCLUDE | | |
| 17 | Description of relevance or appropriateness of studies assembled for assessing the hypothesis to be tested | 2 |
| 18 | The rationale for the selection and coding of data (discouraging, sound clinical principles, or convenience) | 3 |
| 19 | Documentation of how data were classified and coded (e.g., multiple raters, blinding and interrater reliability) | 2 |
| 20 | Assessment of confounding (e.g., comparability of cases and controls in studies where appropriate) | 3 |
| 21 | Assessment of study quality, including blinding of quality assessors, stratification, or regression on possible predictors of study results | 3 |
| 22 | Assessment of heterogeneity | 3 |
| 23 | Description of statistical methods (e.g., complete description of fixed or random effects models, justification of whether the chosen models account for predictors of study results, dose-response models, or cumulative meta-analysis) in sufficient detail to be replicated | 3 |
| 24 | Provision of appropriate tables and graphics | 3 |
| REPORTING OF RESULTS SHOULD INCLUDE | | |
| 25 | Graphic summarizing individual study estimates and the overall estimate | Figure 2-6 |
| 26 | A table giving descriptive information for each study included | Table 1, Supplementary Table 4 |
| 27 | Results of sensitivity testing (e.g., subgroup analysis) | Figure 3, Figure 4, Supplementary Table 3 |
| 28 | Indication of statistical uncertainty of findings | 13, Supplementary Figure 1-2 |
| REPORTING OF DISCUSSION SHOULD INCLUDE | | |
| 29 | Quantitative assessment of bias (e.g., publication bias) | 13 |
| 30 | Justification for exclusion (e.g., exclusion of non-English language citations) | 13 |
| 31 | Assessment of quality of included studies | 13 |
| REPORTING OF CONCLUSIONS SHOULD INCLUDE | | |
| 32 | Conclusions of alternative explanations for observed results | 9-13 |
| 33 | Generalization of the conclusions (e.g., appropriate for the data presented within the domain of the literature review) | 9-13 |
| 34 | Guidelines for future research | 13 |
| 35 | Disclosure of funding source | 13 |

**Supplementary Table 2 The searching strategies of three English databases**

| PubMed |
| --- |
| #1 "Stoke"[MeSH Terms] |
| #2 "Paresis" [MeSH Terms] |
| #3 "Ischemic Attack, Transient" [MeSH Terms] |
| #4 stroke*[Title/Abstract] OR cerebrovascular accident*[Title/Abstract] OR CVA[Title/Abstract] OR apoplexy [Title/Abstract] OR hemipleg*[Title/Abstract] OR paresis[Title/Abstract] OR paretic[Title/Abstract] OR transient ischemic attack[Title/Abstract] OR TIA[Title/Abstract] |
| #5 (cerebral[Title/Abstract] OR cerebellar[Title/Abstract] OR brain*[Title/Abstract] OR vertebrobasilar[Title/Abstract]) AND (infarct*[Title/Abstract] OR ischemi*[Title/Abstract] OR thrombo*[Title/Abstract] OR emboli*[Title/Abstract]) |
| #6 (cerebral[Title/Abstract] OR brain*[Title/Abstract] OR subarachnoid[Title/Abstract]) AND (haemorrhage[Title/Abstract] OR hemorrhage[Title/Abstract] OR haematoma[Title/Abstract] OR hematoma[Title/Abstract] OR bleed*[Title/Abstract]) |
| #7 #1 OR #2 OR #3 OR #4 OR #5 OR #6 |
| #8 "Diabetes Retinopathy" [MeSH Terms] |
| #9 Diabetic Retinopathy[Title/Abstract] OR NPDR[Title/Abstract] OR PDR[Title/Abstract] OR retinopath*[Title/Abstract] |
| #10 #8 OR #9 |
| #11 #7 AND #10 |
| Web of Science |
| #1 TS= (stroke* OR cerebrovascular accident* OR CVA* OR apoplexy OR hempar* OR hemipleg* OR paresis OR paretic OR transient ischemic attack OR TIA*) |
| #2 TS= (cerebral OR cerebellar OR brain* OR vertebrobasilar) |
| #3 TS= (infarct* OR ischemi* OR thrombo* OR emboli*) |
| #4 #2 AND #3 |
| #5 TS= (cerebral OR brain* OR subarachnoid) |
| #6 TS= (haemorrhage OR hemorrhage OR haematoma OR hematoma OR bleed*) |
| #7 #5 AND #6 |
| #8 #1 OR #4 OR #7 |
| #9 TS= (Diabetic Retinopathy OR NPDR OR PDR OR retinopath*) |
| #10 #8 AND #9 |
| Embase |
| #1 'cerebrovascular accident'/exp |
| #2 'brain ischemia'/exp |
| #3 stroke*:ti,ab,kw OR 'cerebrovascular accident*':ti,ab,kw OR cva:ti,ab,kw OR apoplexy:ti,ab,kw OR hemipleg*:ti,ab,kw OR paresis:ti,ab,kw OR paretic:ti,ab,kw OR 'transient ischemic attack':ti,ab,kw OR tia:ti,ab,kw |
| #4 cerebral:ti,ab,kw OR cerebellar:ti,ab,kw OR brain*:ti,ab,kw OR vertebrobasilar:ti,ab,kw |
| #5 infarct*:ti,ab,kw OR ischemi*:ti,ab,kw OR thrombo*:ti,ab,kw OR emboli*:ti,ab,kw |
| #6 #4 AND #5 |
| #7 cerebral:ti,ab,kw OR brain*:ti,ab,kw OR subarachnoid:ti,ab,kw |
| #8 haemorrhage:ti,ab,kw OR hemorrhage:ti,ab,kw OR haematoma:ti,ab,kw OR hematoma:ti,ab,kw OR bleed*:ti,ab,kw |
| #9 #7 AND #8 |
| #10 #1 OR #2 OR #3 OR #6 OR #9 |
| #11 'diabetes retinopathy'/exp |
| #12 'diabetic retinopathy':ti,ab,kw OR npdr:ti,ab,kw OR pdr:ti,ab,kw OR retinopath*:ti,ab,kw |
| #13 #11 OR #12 |
| #14 #10 AND #13 |

**Supplementary Table 3 Meta-regression analysis–strength of covariates in predicting the association between DR and stroke among diabetic patients.**

|  |  | Coeff. | Std. Err. | t | P > \|t\| | 95% C.I. | | Ref. Cat. | F-value | Prob>F | I-squared | Adj. R-squared |
| --- | --- | --- | --- | --- | --- | --- | --- | --- | --- | --- | --- | --- |
| Study design | Retrospective cohort | -0.334 | 0.571 | -0.59 | 0.571 | -1.606 | 0.938 | Prospective cohort | 0.17 | 0.844 | 0.000 | 0.000 |
|  | RCT | -0.231 | 0.632 | 0.37 | 0.722 | -1.639 | 1.176 | Prospective cohort |  |  |  |  |
|  | Prospective cohort | 0.231 | 0.632 | 0.37 | 0.722 | -1.176 | 0. | RCT |  |  |  |  |
| Diabetes type | T2DM | -0.056 | 0.423 | -0.13 | 0.896 | -0.989 | 0.875 | Both | - | - | 0.000 | 0.000 |
|  | Both | 0.056 | 0.423 | 0.13 | 0.896 | -0.875 | 0.989 | T2DM |  |  |  |  |
| Stroke identification method | Medical records | -0.064 | 0.728 | -0.09 | 0.933 | -1.845 | 1.717 | Medical records/self-reported disease history | 0.06 | 0.998 | 0.000 | 0.000 |
|  | Medical records/death certificate/autopsy findings | 0.523 | 2.106 | 0.25 | 0.812 | -4.630 | 5.677 | Medical records/self-reported disease history |  |  |  |  |
|  | Death certificates/medical records/self-reported diagnosis history | 0.264 | 1.184 | 0.22 | 0.831 | -2.634 | 3.164 | Medical records/self-reported disease history |  |  |  |  |
|  | Clinical diagnosis | -0.105 | 0.817 | -0.13 | 0.902 | -2.105 | 1.895 | Medical records/self-reported disease history |  |  |  |  |
|  | Clinical diagnosis/autopsy | 0.164 | 0.987 | 0.17 | 0.873 | -2.251 | 2.580 | Medical records/self-reported disease history |  |  |  |  |
|  | Medical records/death certificate | -0.376 | 1.265 | -0.30 | 0.776 | -3.474 | 2.721 | Medical records/self-reported disease history |  |  |  |  |
|  | Medical records/self-reported disease history | -0.264 | 1.184 | -0.22 | 0.831 | -3.164 | 2.634 | Death certificates/ medical records/self-reported diagnosis history |  |  |  |  |
